# circRNA hsa_circ_0005909 Predicts Poor Prognosis and Promotes the Growth, Metastasis, and Drug Resistance of Non-Small-Cell Lung Cancer via the miRNA-338-3p/SOX4 Pathway

**DOI:** 10.1155/2021/8388512

**Published:** 2021-08-06

**Authors:** He-mei Song, Dan Meng, Jin-ping Wang, Xiao-yan Zhang

**Affiliations:** ^1^Department of Pharmacy, The Affiliated Lianyungang Hospital of Xuzhou Medical University, The First People's Hospital of Lianyungang, Lianyungang, Jiangsu, China; ^2^Department of General Surgery, Qingdao Eighth People's Hospital, Qingdao, Shandong, China; ^3^Department of Gynaecology, Qingdao Eighth People's Hospital, Qingdao, Shandong, China; ^4^Department of Pharmacy Intravenous Admixture, Weifang People's Hospital, Weifang, Shandong, China

## Abstract

**Background:**

Circular RNAs (circRNAs) are powerful factors in regulating various cancer behaviors. It has been manifested in previous researches that circular RNA hsa_circ_0005909 (circ_0005909) exhibits a regulatory function in osteosarcoma. However, there are no other studies on whether circ_0005909 displays potential functions on the progression of non-small-cell lung cancer (NSCLC).

**Methods:**

RT-PCR was applied to examine the expression of circ_0005909 in NSCLC. To study the specific behaviors of NSCLC cells after circ_0005909 knockdown, cell counting kit-8 (CCK-8) assays, colony formation assays, Transwell assays, and xenograft tumor model assays were conducted. Bioinformatics and luciferase reporter assays were employed to study the association among circ_0005909, miRNA-338-3p, and SOX4.

**Results:**

In this research, our group firstly showed that circ_0005909 expressions were distinctly increased in NSCLC specimens and cell lines. Clinical studies revealed that high circ_0005909 expressions were associated with poor prognosis of NSCLC patients. Functionally, knockdown of circ_0005909 was observed to suppress the proliferation, metastasis, and drug resistance of NSCLC cells. In the terms of mechanism, circ_0005909 could act as a sponge of miRNA-338-3p, and miRNA-338-3p could target SOX4. In addition, miRNA-338-3p inhibitors reversed the suppressor ability of circ_0005909 silence on NSCLC behaviors.

**Conclusions:**

circ_0005909 promoted the progression of NSCLC via the modulation of the miRNA-338-3p/SOX4 axis, which may be a therapeutic target for NSCLC.

## 1. Introduction

As one of the most frequent malignancies in the world, lung cancer is rapidly becoming the main cause of tumor-related death nowadays [1, 2]. Non-small-cell lung cancer (NSCLC) accounts for eighty percent of novel lung cancer cases [3]. Up to date, many patients were at advanced stages when diagnosed, which resulted in a very disadvantageous situation that the prognosis of NSCLC patients is still dissatisfying, in spite of more and more developments in clinical and experimental oncology [4, 5]. Thus, to improve the prevention, treatments, and survival rates of NSCLC, further studies of the detailed knowledge of the mechanisms underlying NSCLC progression were necessary.

Circular RNAs (circRNAs), characterized by closed loop structures without 5′ caps and 3′ poly(A) tails, are a class of noncoding RNAs [6]. circRNAs are derived mainly from introns or exons, while exonic circRNAs were mainly expressed in the cytoplasm and exhibit better stabilities than linearity RNAs [7]. In recent years, more and more circRNAs have been demonstrated to be involved in the regulation of gene expressions via sponging miRNAs [8]. Then, a growing number of studies suggest that the abnormal levels of circRNAs are significantly involved in carcinogenesis and progression of various tumors, including NSCLC [9, 10]. A growing number of researches have revealed that circRNAs may function as antioncogenes or tumor promotors in several types of tumors through competitively binding to miRNAs [11, 12]. Although more and more circRNAs have been identified to display a dysregulated level in NSCLC, their function and the related molecular mechanisms in NSCLC progression remained largely unclear.

Circular RNA hsa_circ_0005909 (circ_0005909), a newly identified circRNA with a length of 371 nucleotides, originated from the reverse splicing of XPR1 mRNAs. Recently, the distinct overexpression of circ_0005909 was reported in osteosarcoma, and its oncogenic roles were also demonstrated in cellular experiments [13, 14]. However, the expression and functions of circ_0005909 in other tumors have not been investigated. Here, our group firstly provided evidence that circ_0005909 expressions were increased in NSCLC. Moreover, we further explored the effects of circ_0005909 in NSCLC behaviors.

## 2. Materials and Methods

### 2.1. Clinical Specimens

A total of 102 paired NSCLC specimens and the matched noncancer specimens were obtained from NSCLC patients undergoing surgery at the Weifang People's Hospital between July 2013 and March 2016. Based on histopathological evaluation, patients with NSCLC were diagnosed. The protocols used in this study were approved by the Ethical Review Committees of Weifang People's Hospital, and written informed consent was provided from all patients.

### 2.2. Cell Culture and Transfection

Human NSCLC cell lines (H460, A549, Calu-3, and SK-MES-1) and normal human bronchial epithelial cells (NHBE) were provided by Jihe Technology (Shanghai, China). These cells were cultured in DMEM or RPMI 1640 medium (Lonza, Zheping Biology, China), containing 10% fetal bovine serum (FBS, MedChemExpress, USA) at 37°C with 5% CO_2_ in a humidified incubator.

For the dysregulation of various factors, small hairpin RNA of circ_0005909 (sh-circ_0005909), miRNA-338-3p inhibitors (5′-GCAAAAAUUAGUGUGCGCCACC-3′), miRNA-338-3p mimics (5′-UUUGAGCAGCACUCAUUUUUGC-3′), and their negative controls (5′-CAGUACUUUCAGUGCCAUCACAC-3′) were provided by Hewu Company (Shanghai, Fengxian, China). To increase circ_0005909 levels, full length circ_0005909 was cloned into a modified LV003 lentiviral vector (Hewu Company, Shanghai, Fengxian, China). An empty vector served as a control. Based on the product guide, Lipofectamine 2000 (Invitrogen, Hangzhou, Zhejiang, China) was applied for the transient transfection into A549 and H460 cells. Cells were harvested at 48 h posttransfection.

### 2.3. Bioinformatics Analysis

To explore the potential networks among circRNAs, miRNAs, and target mRNAs, CircInteractome was applied to predict miRNA-338-3p binding sites to the circ_0005909 and TargetScan was applied for the prediction of the potential miRNA-338-3p binding sites to 3′-UTR of SOX4.

### 2.4. RNA Extraction and qRT-PCR

For the collection of total RNAs, the TRIzol Reagent (Invitrogen, Pudong, Shanghai, China) was used based on product guides. PrimeScript RT Reagent Kit (Bio-Rad, Aiyan Biology, Minhang, China) was applied to synthesize complementary DNA. By the use of a PrimeScript™ RT Reagent Kit (Bio-Rad, Aiyan Biology, Minhang, China), one microgram of total RNA was reverse transcribed in a final volume of 25 *μ*l. The relative levels of lncRNAs and mRNAs were determined by RT-PCR, which was carried out by the use of the ABI Power SYBR Green PCR Master Mix (Applied Biosystems, China). U6 and GAPDH were applied as internal controls. The relative expressions of the target genes were assessed via 2^−ΔΔCt^ methods. All primer sequences are listed in Table 1.

### 2.5. RNase R Treatments

Total RNAs (15 *μ*g) were incubated with or without 4 U·*μ*g^−1^ of RNase R (Geneseed, Jisai Biology, Kexuecheng, Guandong, China). After incubation at 37°C for 20 min, the RNeasy MinElute Cleaning Kit (Qiagen, Nanjing, China) was used to purify the collected RNAs. Finally, RT-PCR assessed the expressions of RNAs.

### 2.6. Cell Counting Kit-8 (CCK-8) Assays

Cells were resuspended and plated into 96-well plates. They were cultured for 24 h, 48 h, 72 h, and 96 h, respectively, and then added with the Cell Counting Kit-8 reagent. Two hours later, the optical density value of each well was measured.

### 2.7. Colony Formation Assay

A549 and H460 cells were cultured for 14 days in 6-well plates at a density of 500/well. Subsequently, cells were fixed in 4% paraformaldehyde (Yubo, Shanghai, China) and stained with 0.1% crystal violet (TargetMol, Jingan, Shanghai, China). Finally, the collected cells were counted manually.

### 2.8. EdU Staining

Cellular proliferation was also determined using EdU assays. A549 and H460 were seeded in 48-well plates, followed by EdU medium (Guyan, Shanghai) which was used to incubate the above cells. The EdU staining reagents were applied to color cells. After washing in PBS three times, A549 and H460 cells were cultured in DAPI solution. The images were obtained by inverted fluorescence microscopy.

### 2.9. Transwell Assays

On the upper Transwell chambers,5 × 10^4^transfected cells were seeded. To the lower chamber, our group added medium with 20% FBS. Matrigel (FS-79049, Fusheng Biology, Pudong, Shanghai, China) was used to precoat the membrane. On the top chamber, 1.0 × 10^5^ cells were added. After one day, the chamber was fixed with 4% paraformaldehyde and stained using 0.05% crystal violet (Beyotime, Haidian, Beijing, China). The invasive cells were imaged and counted.

### 2.10. Luciferase Reporter Assay

The 3′-UTR of SOX4 was amplified and cloned downstream of the pGL3/Luciferase vector (SOX-Wt). Then, the mutant 3′-UTR of SOX4 was amplified and cloned downstream of the pGL3/Luciferase vector (SOX-Mut). circ_0005909 wild-type (circ_0005909_Wt) and mutant (circ_0005909_Mut) reporter vectors were constructed and inserted into pGL3. A549 and H460 cells were cotransfected into the luciferase reporter vectors and miR-NC or miR-338-3p. After one day, cells were lysed. Dual-Luciferase Reporter System (Promega, Haidian, Beijing, China) was applied to examine relative luciferase activities.

### 2.11. Subcellular Fractionation

Based on the manufacturer's directions, the PARIS kit (Life Technologies, Haidian, Beijing, China) was applied to perform nuclear and cytosolic fraction separation.

### 2.12. RNA Pull-Down Assay

The commercially synthesized biotin-labeled miRNA-338-3p was purchased from Chenhua Biology (Hangzhou, Zhejiang, China) and transfected into A549 and H460 cells for 48 h. Then, the Dynabeads M-290 Streptavidin (Sigma-Aldrich, Nanjing, Jiangsu, China) was applied to incubate the cell lysate based on the product guide. For the purification of the interacted RNA complex, TRIzol was used. The levels of circ_0005909 were determined using RT-PCR. Three independent experiments were performed.

### 2.13. Western Blot Assays

To extract total protein from the NSCLC tissues and cell lines, RIPA buffer (MedChemExpres, Pudong, Shanghai, China) was used. A BCA Protein Assay Kit (Beyotime) was used for the quantification of protein concentrations. The specific procedures of the western blot were described in the previous study. The primary antibodies against SOX4 (1 : 1000, Guduo Biology, Pudong, Shanghai, China) was used in this experiment. GAPDH was used as a control.

### 2.14. In Vivo Tumor Growth Assay

A549 cells were transfected with sh-circ_0005909 or sh-NC. Cells were injected subcutaneously into BALB/c nude mice (*n* = 6/group, females, 5-6 weeks old; Saiye Biology, Suzhou, Jiangsu, China). The Animal Care and Use Committee of Weifang People's Hospital has approved the animal experiments. At the indicated times, tumor volumes were examined, and the following equation was used for the calculation: volume = (length × width^2^)/2. After seven weeks, Mice were killed, and the collected tumors were weighed and harvested for subsequent study.

### 2.15. Statistical Analysis

All data were analyzed using the SPSS 15.0 software. To explore the differences between two groups (or >two groups), Student's *t*-test or one-way analysis of variance (ANOVA) followed by the LSD post hoc test was conducted. The differences of categorical factors were determined using the Chi-squared test. The Kaplan-Meier method and the log-rank test were applied for the survival assays. The Cox regression model was conducted for univariate and multivariate assays. Values of *p* < 0.05 were considered significant.

## 3. Results

### 3.1. An Increased Expression of circ_0005909 Was Observed in NSCLC

**Figure 1:**
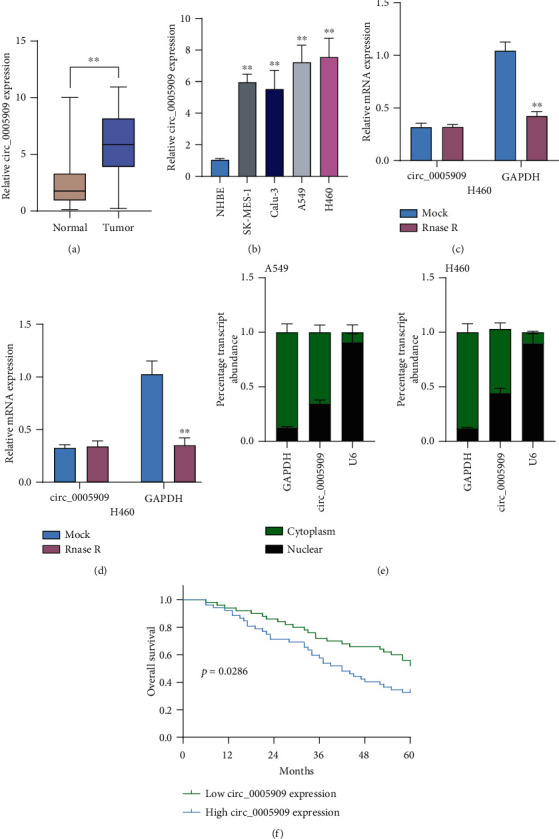
circ_0005909 expression was upregulated in NSCLC. (a) The expressions of circ_0005909 in 15 pairs of NSCLC samples and matched nontumor samples via RT-PCR. (b) RT-qPCR assay examined the circ_0005909 expressions in four NSCLC cell lines and NHBE cells. (c, d) Relative circ_0005909 expression and GAPDH in A549 and H460 cells treated with RNase R. (e) Relative SNHG1 expression levels in nuclear and cytosolic fractions of H460 and A549 cells. (f) Survival assays of 102 NSCLC patients based on the mean expression of circ_0005909 (^∗∗^*p* < 0.01).

Firstly, we performed RT-PCR to examine whether circ_0005909 was abnormally expressed in NSCLC. As presented in Figure 1(a), circ_0005909 expression in NSCLC specimens was distinctly higher than those in the matched noncancer specimens. An increased level of circ_0005909 was demonstrated in four NSCLC cell lines compared with NHBE cells (Figure 1(b)). To study the circular characteristics of circ_0005909, we performed RNase R treatments, finding that the circ_0005909 expressions did not change while GAPDH levels were distinctly decreased after the treatments of RNase R in both A549 and H460 cells (Figures 1(c) and 1(d)). Using fluorescence subcellular fractionation, we observed that a larger proportion of circ_0005909 was in the cytoplasm (Figure 1(e)).

### 3.2. High-Expression Level of circ_0005909 Predicts Poor Prognosis in NSCLC Patients

The circ_0005909 expression levels were classified as high or low in relation to the median value. A chi-square test indicated that high circ_0005909 expression was associated with tumor node metastasis (TNM) stage (*p* = 0.023) and distant metastasis (*p* = 0.028) (Table 2). For further exploration of the prognostic values of circ_0005909 dysregulation in NSCLC specimens, our group conducted the Kaplan-Meier survival assays, finding that patients with high expression of circ_0005909 had shorter overall survival as compared with the circ_0005909-low group (*p* = 0.0286, Figure 1(f)). More importantly, multivariate analysis demonstrated that high circ_0005909 expression was independently associated with overall survival (HR = 2.569; 95% CI: 1.138-3.667; *p* = 0.031; Table 3).

### 3.3. The Antioncogenic Roles of circ_0005909 Silence on the Proliferation and Metastasis of NSCLC Cells

**Figure 2:**
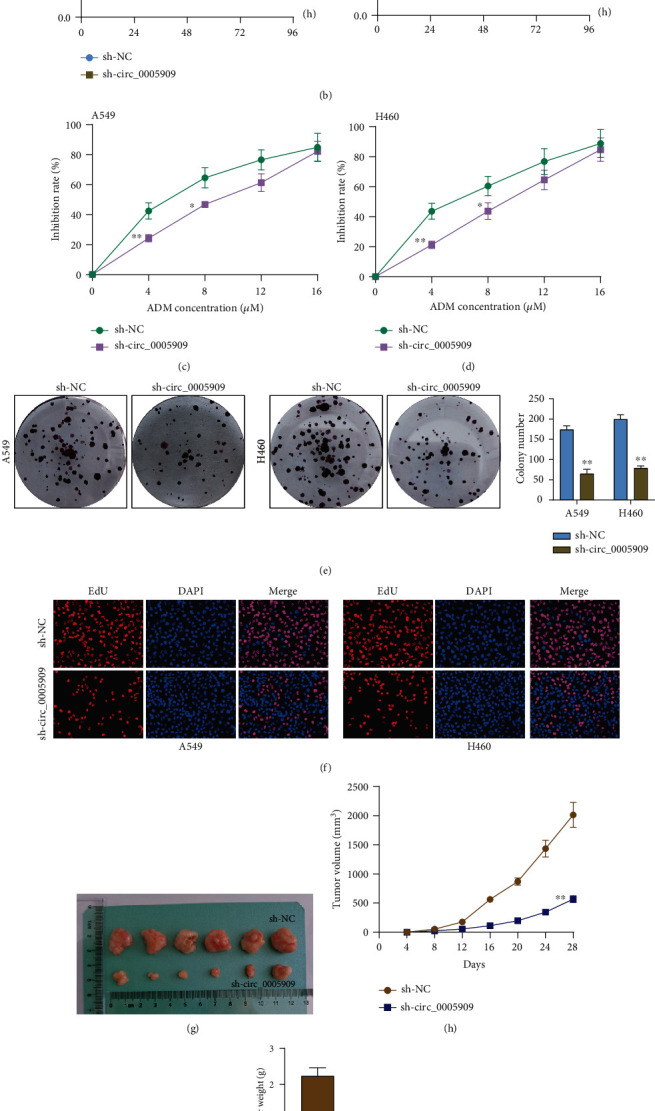
The function of circ_0005909 knockdown on tumor biological behaviors. (a) RT-PCR determined the levels of circ_0005909 in NSCLC cells transfected with sh-circ_0005909 or sh-NC. (b) CCK-8 assays after circ_0005909 knockdown. (c, d) The effects of ADM treatments on the inhibition of A549 and H460 cells. (e, f) Colony formation assays and EdU assays were performed to detect cell proliferation. (g) Tumors from the sh-NC or sh-circ_0005909 group are shown. (h, i) Volume and weight of tumors obtained from the sh-NC or sh-circ_0005909 group are displayed. (j) Transwell assays of NSCLC cells transfected with sh-circ_0005909 or sh-NC (^∗∗^*p* < 0.01 and ^∗^*p* < 0.05).

To study the potential functions of circ_0005909 in NSCLC behaviors, we downregulated the expressions of circ_0005909. RT-PCR assays demonstrated the transfection efficiency of sh-circ_0005909 in both A549 and H460 cells. However, the expression of XPR1 mRNA remained unchanged after the transfection of sh-circ_0005909 (Figure 2(a)). The growth curves from CCK-8 assays suggested that circ_0005909 silence distinctly suppressed cell proliferation and colony formation in the A549 and H460 cell lines (Figure 2(b)). We also explored the effect of circ_0005909 on chemotherapy resistance of NSCLC cells, finding that inhibition rate was suppressed with the increase of concentration of ADM, and silence of circ_0005909 decreased the suppressor effects of ADM on A549 and H460 cells (Figures 2(c) and 2(d)). Besides, the colony formation and EdU assays demonstrated that downregulation of circ_0005909 led to reduced cellular growth (Figures 2(e) and 2(f)). Further in vivo assays revealed that circ_0005909 knockdown decreased tumor volume and weight (Figures 2(g)–2(i)). Moreover, we explored whether circ_0005909 dysregulation may influence the potential metastasis of A549 and H460 cells. Transwell assays revealed that invasive A549 and H460 cells were distinctly decreased after the knockdown of circ_0005909 (Figure 2(j)).

### 3.4. Circ_0005909 Sponges miRNA-338-3p in NSCLC

**Figure 3:**
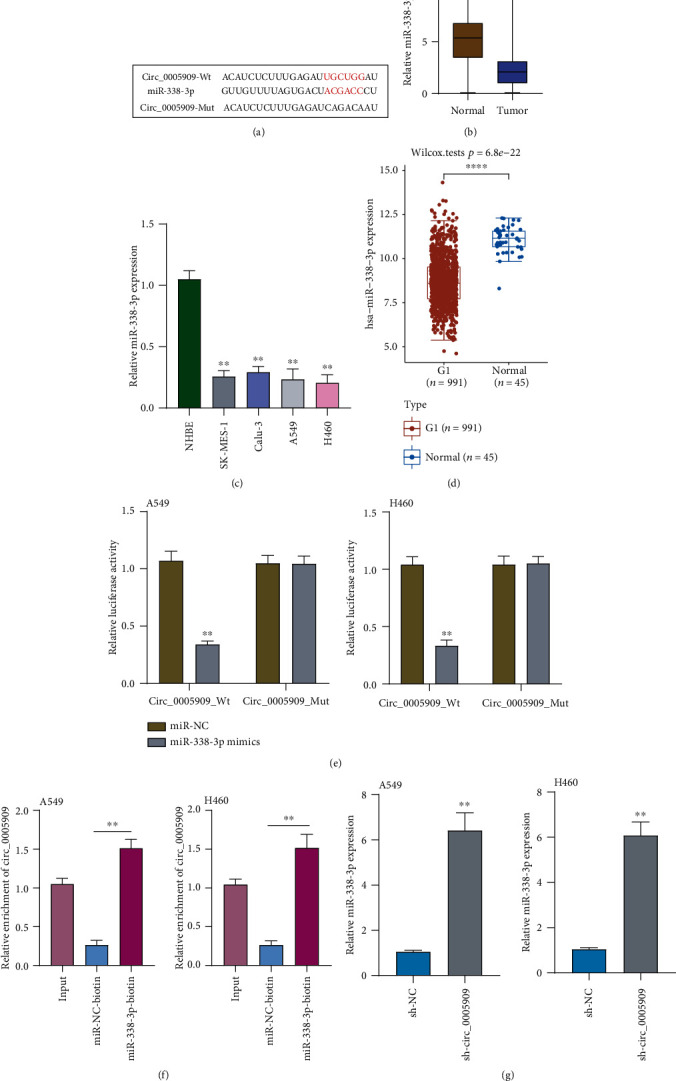
circ_0005909 regulates miRNA-338-3p in NSCLC cells. (a) Schematic graph of the putative binding sites of miRNA-338-3p in the circ_0005909. (b) The expressions of miRNA-338-3p in tumor samples by RT-PCR. (c) Increased miRNA-338-3p expression is shown in four NSCLC cells, determined by RT-PCR. (d) miRNA-338-3p expressions are shown using TCGA datasets. (e) Luciferase reporter assay shows that the luciferase activity of either circ_0005909-WT was inhibited by miRNA-338-3p mimics. (f) Enrichment of circ_0006282 using the RNA pull-down experiment. (g) RT-PCR determined the levels of miRNA-338-3p in A549 and H460 cells after the transfection (^∗∗^*p* < 0.01).

**Figure 4:**
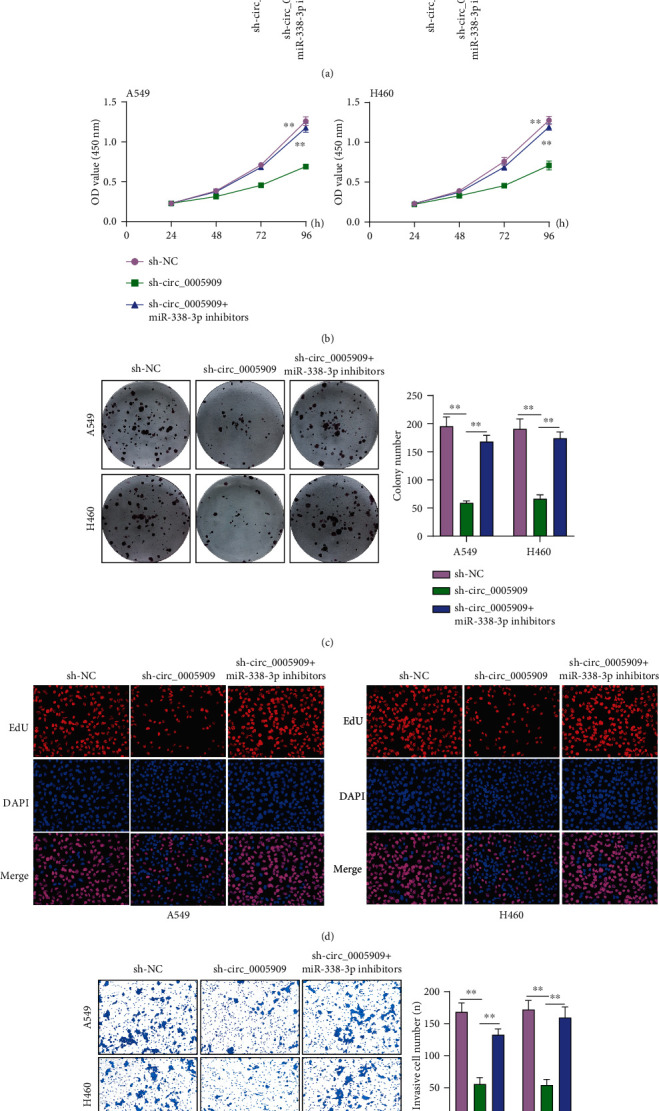
The oncogenic roles of circ_0005909 in NSCLC through sponging miRNA-338-3p. (a) The expressions of miRNA-338-3p in A549 and H460 cells transfected with circ_0005909+miRNA-338-3p inhibitors, sh-circ_0005909, or sh-NC using RT-PCR. (b) CCK-8 assay of cell viability in NSCLC cells transfected with sh-circ_0005909 or circ_0005909+miRNA-338-3p inhibitor, or sh-NC. (c) Colony formation in A549 and H460 cells transfected with sh-NC, sh-circ_0005909, or circ_0005909+miRNA-338-3p inhibitors. (d) Edu assays in A549 and H460 cells transfected with sh-circ_0005909, circ_0005909+miRNA-338-3p inhibitors, or sh-NC. (e) A549 and H460 cell invasion using Transwell assays (^∗∗^*p* < 0.01).

Many researches have suggested that cytoplasmic circRNAs might regulate the expressions of various genes via functioning as miRNA sponges [15]. We have shown that circ_0005909 was enriched in the cytoplasm, suggesting that it may exhibit its function via acting as a ceRNA. Using CircInteractome software, we observed that miRNA-338-3p may have a high probability of binding to circ_0005909 (Figure 3(a)). Previously, several studies showed that miRNA-338-3p served as a tumor suppressor, suggesting the probability that circ_0005909 may promote NSCLC progression via sponging miRNA-338-3p [16, 17]. So, we chose miRNA-338-3p for further study. RT-PCR suggested that miRNA-338-3p expressions were distinctly decreased in both tumor specimens and cell lines (Figures 3(b) and 3(c)). The Cancer Genome Atlas (TCGA) datasets also showed that miRNA-338-3p expressions were downregulated in NSCLC specimens (Figure 3(d)). miRNA-338-3p mimics distinctly decreased the luciferase activity of circ_0005909-WT (Figure 3(e)). Moreover, circ_0005909 was enriched in the miRNA-338-3p group based on RNA pull-down assays (Figure 3(f)). In addition, the expressions of miRNA-338-3p were distinctly increased in sh-circ_0005909-transfected A549 and H460 cells (Figure 3(g)). Then, rescue experiments were applied to study the direct roles between circ_0005909 and miRNA-338-3p. RT-PCR indicated that miRNA-338-3p silence distinctly reversed the promotion of circ_0005909 silence on the expressions of miRNA-338-3p in A549 and H460 cells (Figure 4(a)). CCK-8 assays revealed that the silence of circ_0005909 decreased the proliferation of A549 and H460 cells, and that cellular viabilities were distinctly increased in the sh-circ_0005909+miRNA-338-3p inhibitor group compared with the sh-circ_0005909 group (Figure 4(b)). In colony assays and Edu assays, similar results were also observed (Figures 4(c) and 4(d)). Transwell assays indicated that cellular invasions were suppressed by sh-circ_0005909 and restored dual suppression of circ_0005909 and miRNA-338-3p (Figure 4(e)). Based on our findings, circ_0005909 may promote NSCLC progression through sponging miRNA-338-3p.

### 3.5. Circ_0005909 Promotes SOX4 Expressions through Sponging miRNA-338-3p

**Figure 5:**
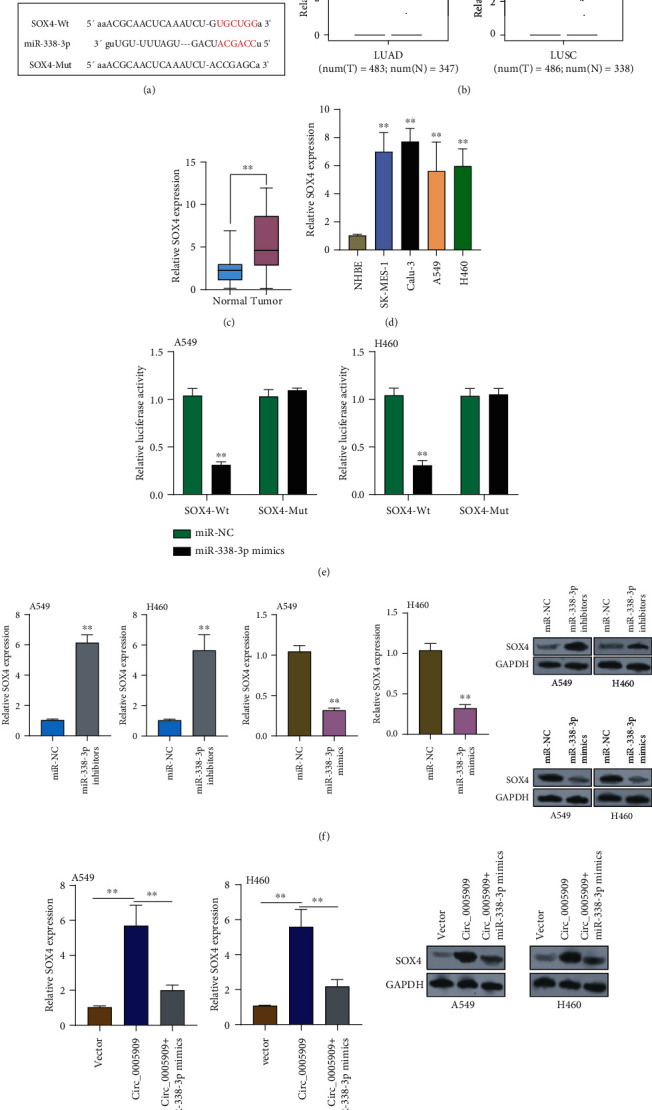
SOX4 was a target of miRNA-338-3p. (a) The binding site of SOX4 3′-UTR for miRNA-338-3p. (b) By searching TCGA datasets, the dysregulation of SOX4 was determined. (c) The levels of SOX4 in our cohort. (d) RT-PCR assays for the levels of SOX4 in four NSCLC cell lines and NHBE cells. (e) Luciferase reporter assay was used to measure the targeting relationship between miRNA-338-3p and SOX4. (f) RT-PCR assays and western blot assays for the levels of SOX4 in A549 and H460 cells transfected with miR-NC, miRNA-338-3p inhibitors, or miRNA-338-3p mimics. (g) SOX4 expression was determined in H460 and A549 cells transfected with vector, circ_0005909, or circ_0005909+miRNA-338-3p mimics (^∗∗^*p* < 0.01).

Using an online tool (TargetScan), we observed that SOX4 may be a potential target of miRNA-338-3p (Figure 5(a)). Based on TCGA datasets, we observed that SOX4 expression was distinctly upregulated in lung cancer specimens (Figure 5(b)). The similar overexpression of SOX4 was also observed in our cohort (Figure 5(c)). In addition, RT-PCR also showed that SOX4 expressions were increased in four NSCLC cells compared with NHBE cells (Figure 5(d)). Luciferase reporter assays revealed that miRNA-338-3p mimics decreased the relative luciferase activity of SOX4-WT (Figure 5(e)). Furthermore, the silence of miRNA-338-3p resulted in the distinct upregulation of SOX4, while miRNA-338-3p overexpression exhibited an opposite result (Figure 5(f)). Finally, the rescue experiments revealed that miRNA-338-3p upregulation reversed the distinct promotion of circ_0005909 overexpression on the expressions of SOX4 (Figure 5(g)).

## 4. Discussion

The identification of novel modulators in NSCLC progression was very important for the improvements of targeted therapies [18]. Many studies have demonstrated that circRNAs exhibit regulatory functions in miRNA inhibitions and tumorigenesis, including NSCLC [19]. For instance, circ_0074027 was highly expressed in NSCLC, and its overexpression distinctly promoted the metastasis of NSCLC cells via increasing RHOA expression through sponging miRNA-2467-3p [20]. circNDUFB2 was shown to exhibit a low level in NSCLC, and its forced expression distinctly resulted in the ability to suppress the growth and invasion of NSCLC cells via destabilizing IGF2BPs and activating antitumor immunity [21]. These findings encouraged us to further identify other functional circRNAs. In this research, our group discriminated a novel NSCLC-related circRNA, circ_0005909, which was distinctly overexpressed in NSCLC specimens. Clinical assays revealed that high circ_0005909 expression indicated a poor prognosis of NSCLC patients. Functionally, we observed that silence of circ_0005909 suppressed the proliferative and metastatic abilities of NSCLC cells as well as drug resistance, indicating circ_0005909 might be a therapeutic target of NSCLC. Previously, the tumor-promotive roles of circ_0005909 in osteosarcoma were also reported [22]. Thus, whether there was a cross-talk for the efficiency and specificity of circ_0005909 needed to be further studied.

For the exploration of mechanism assays, a large number of researches have demonstrated that circRNAs modulate tumor progression via serving as miRNA sponges [23, 24]. In other words, circRNAs can bind miRNAs to suppress their expressions and competitively result in the suppression of the interreaction between miRNAs and their potential targeting genes, thereby modulating tumor progression. For instance, circ_0110757 was reported to increase temozolomide resistance in neuroblastoma via sponging miRNA-1298-5p [25]. Circular RNA RHOT1 promoted metastasis and suppressed ferroptosis via the miR-106a-5p/STAT3 axis in breast cancer [26]. Previously, circ_0005909 was shown to sponge miRNA-338-3p and miR-936 in osteosarcoma, thus promoting the progression of osteosarcoma [13, 22]. These findings suggested that circ_0005909 is a potential therapeutic target for osteosarcoma. However, whether circ_0005909 also exhibited its effects on NSCLC progression via sponge miRNAs has not been investigated. Our study proved that circ_0005909 binds with miRNA-338-3p to decrease its expressions, suggesting a novel circRNA/miRNA axis in NSCLC cell progression. In recent years, high expressions of miR-338-3p and its effects on promoting the growth and invasion of tumor cells have been frequently reported in many types of tumors, including NSCLC [16, 27]. Here, our group further reported that the suppressor functions of circ_0005909 knockdown on the proliferative and metastatic abilities of NSCLC cells could be reversed by miRNA-338-3p inhibition, which indicated that circ_0005909 may sponge miRNA-338-3p to promote the uncontrollable behaviors of NSCLC cells.

SOX4 is a member of the SOX (SRY-related HMG box) gene family consisting of transcription factors involved in differentiations of cells and organs, and the progression of various types of tumors [28, 29]. Many researches have demonstrated that SOX4 expressions are distinctly upregulated in over 22 malignant cancers and serve as oncogenes in malignant phenotypes [30, 31]. Importantly, several studies have provided evidence that SOX4 expression was increased in NSCLC and promoted the tumor growth and migration of NSCLC [32, 33]. In this study, we also showed a high level of SOX4 in NSCLC. Interestingly, bioinformatics assays revealed that miRNA-338-3p could target SOX4 3′-UTR. Based on the data of RNA pull-down experiments and luciferase reporter assays, we observed that they could target and bind, and miRNA-338-3p could suppress SOX4 expressions. On the other hand, rescue experiments demonstrated that overexpression of miRNA-338-3p reversed the obvious suppressions of circ_0005909 overexpression on levels of SOX4. These findings suggested that circ_0005909, as a ceRNA, promoted the growth, invasion, and metastasis of NSCLC through the modulation of the miRNA-338-3p/SOX4 signal axis.

In sum, the current data depicted a circ_0005909/miRNA-338-3p/SOX4 axis in NSCLC and implied that circ_0005909 promoted NSCLC progression. Therefore, circ_0005909 was a candidate biomarker and therapeutic target for NSCLC patients.

## Figures and Tables

**Table 1:** Primer sequences.

Primer	Sequences (5′-3′)
Hsa_circ_0005909: forward	GTATCCACTTGCCCTTTA
Hsa_circ_0005909: reverse	TTACTCCAGCCTGTCTC
miRNA-338-3p: forward	ATCCAGTGCGTGTCGTGG
miRNA-338-3p: reverse	TGCTTCCAGC ATCAGTGAT
XPR1: forward	TGGTGTTACTACGCTGCGAC
XPR1: reverse	CACTGAAGGCCAGTTTAAGGTC
SOX4: forward	GACCTGCTCGACCTGAACC
SOX4: reverse	CCGGGCTCGAAGTTAAAATCC
GAPDH: forward	GGAGCGAGATCCCTCCAAAAT
GAPDH: reverse	GGCTGTTGTCATACTTCTCATGG
U6: forward	CTCGCTTCGGCAGCACA
U6: reverse	AACGCTTCACGAATTTGCGT

**Table 2:** Correlation of circ_0005909 expression with clinicopathological features of NSCLC.

Clinicopathological features	Number of cases	circ_0005909 expression		*p* value
		High	Low	
Age (years)				0.988
<60	57	28	27	
≥60	45	24	23	
Gender				0.241
Male	59	33	26	
Female	43	19	24	
Tumor size (cm)				0.217
<3	59	27	32	
≥3	43	25	18	
TNM stage				0.023
I+II	62	26	36	
III+IV	40	26	14	
Distant metastasis				0.028
Yes	33	22	11	
No	69	30	29	

**Table 3:** Cox regression analysis of factors associated with overall survival in 102 NSCLC patients.

Variables	Univariate analysis		Multivariate analysis	
	HR (95% CI)	*p* value	HR (95% CI)	*p* value
Age	0.786 (0.323-1.233)	0.452	—	—
Gender	0.653 (0.443-1.093)	0.391	—	—
Tumor size	1.023 (0.673-1.453)	0.294	—	—
TNM stage	2.986 (1.342-4.472)	0.014	2.765 (1.217-3.986)	0.029
Distant metastasis	3.237 (1.438-5.482)	0.003	2.986 (1.283-4.776)	0.007
circ_0005909 expression	2.738 (1.338-4.375)	0.016	2.569 (1.138-3.667)	0.031

## Data Availability

The data used to support the findings of the present study are available from the corresponding author upon reasonable request.
